# Supplementary material for: Anthropogenic shift towards higher risk of flash drought over China
Source: Nat Commun. 2019 Oct 11;10:4661. doi: 10.1038/s41467-019-12692-7 (PMC6789133; doi:10.1038/s41467-019-12692-7)
Supplement: Supplementary file 1 — Supplementary Information [file 41467_2019_12692_MOESM1_ESM.doc]

Supplementary Information for

**Anthropogenic shift towards higher risk of flash drought over China**

by Yuan et al.

**Supplementary Figures**

**
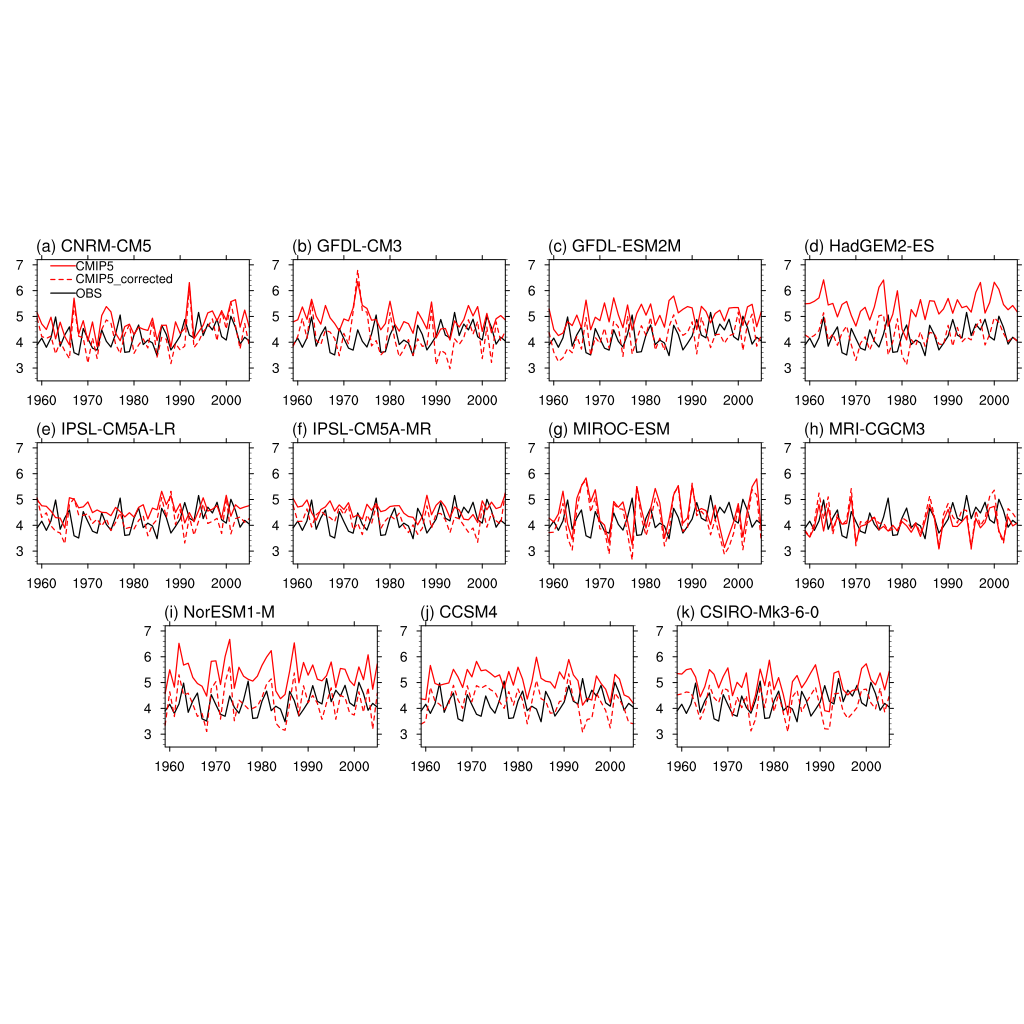
**

**Supplementary Figure 1.** Comparison of original and bias-corrected CMIP5 July mean precipitation (mm/day) averaged over China during 1959-2005. Black lines are observations, solid and dashed red lines are original and bias-corrected CMIP5 precipitation. The equidistant CDF matching method (see Methods for details) was used for bias correction of monthly model data.


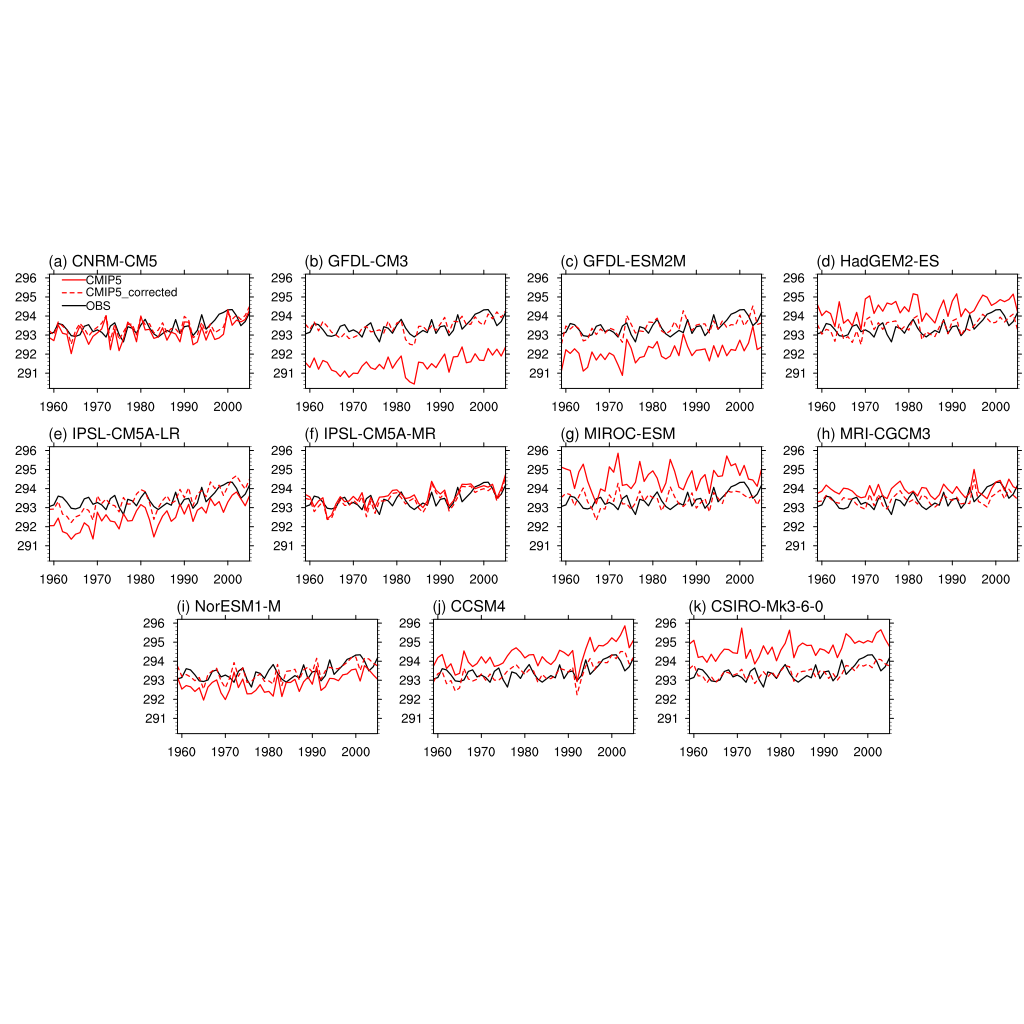


**Supplementary Figure 2.** The same as Supplementary Figure 1, but for surface air temperature (K).


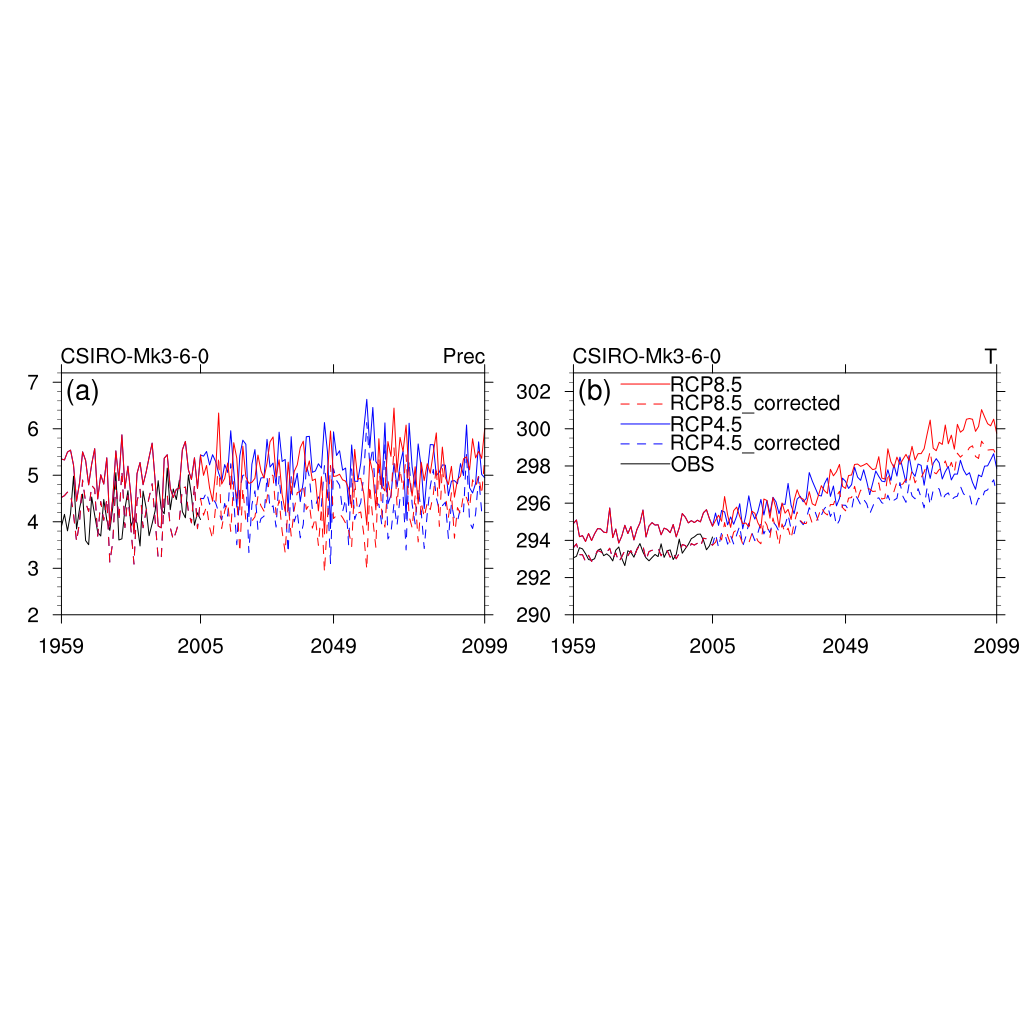


**Supplementary Figure 3.** An example of CMIP5 model data with or without bias correction for historical and future July precipitation (a; mm/day) and temperature (b; mm/day) averaged over China under ALL forcings (1959-2005) and RCP4.5 and RCP8.5 scenarios (2006-2099).


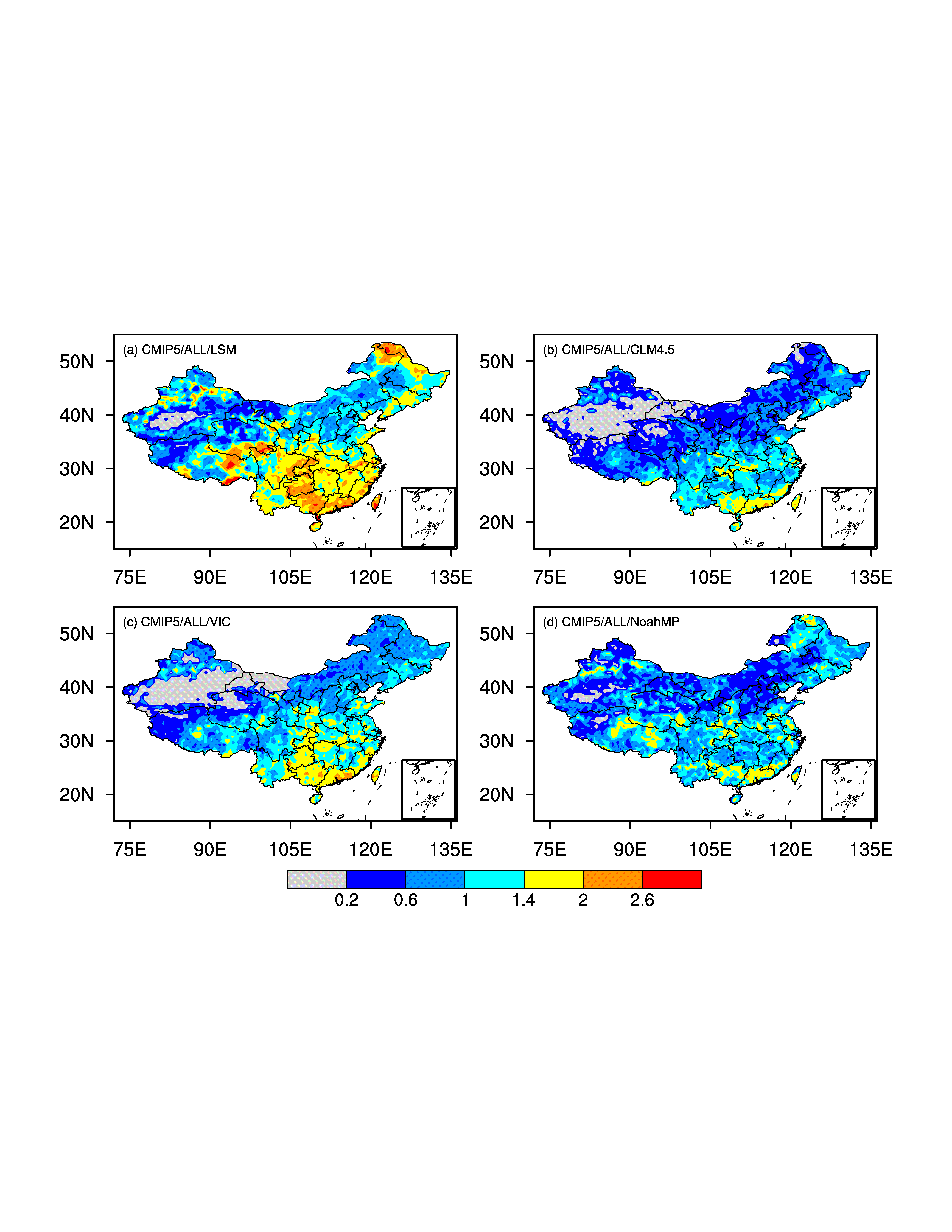


**Supplementary Figure 4.** Uncertainty ranges (standard deviations) of the frequency of flash drought events (events/decade) from CMIP5/ALL/LSM simulations. There are 29, 11, 11 and 7 realizations for ALL/LSMs (a), ALL/CLM4.5 (b), ALL/VIC (c) and ALL/NoahMP (d) simulations, respectively (see Supplementary Table 1). All statistics are based on the data during 1961-2005. Maps were created by using the NCAR Command Language (Version 6.3.0) [Software]. (2016). Boulder, Colorado: UCAR/NCAR/CISL/TDD. <http://dx.doi.org/10.5065/D6WD3XH5>. And the maps were updated with a database provided by <https://coding.net/u/huangynj/p/NCL-Chinamap/git/tree/master/database>


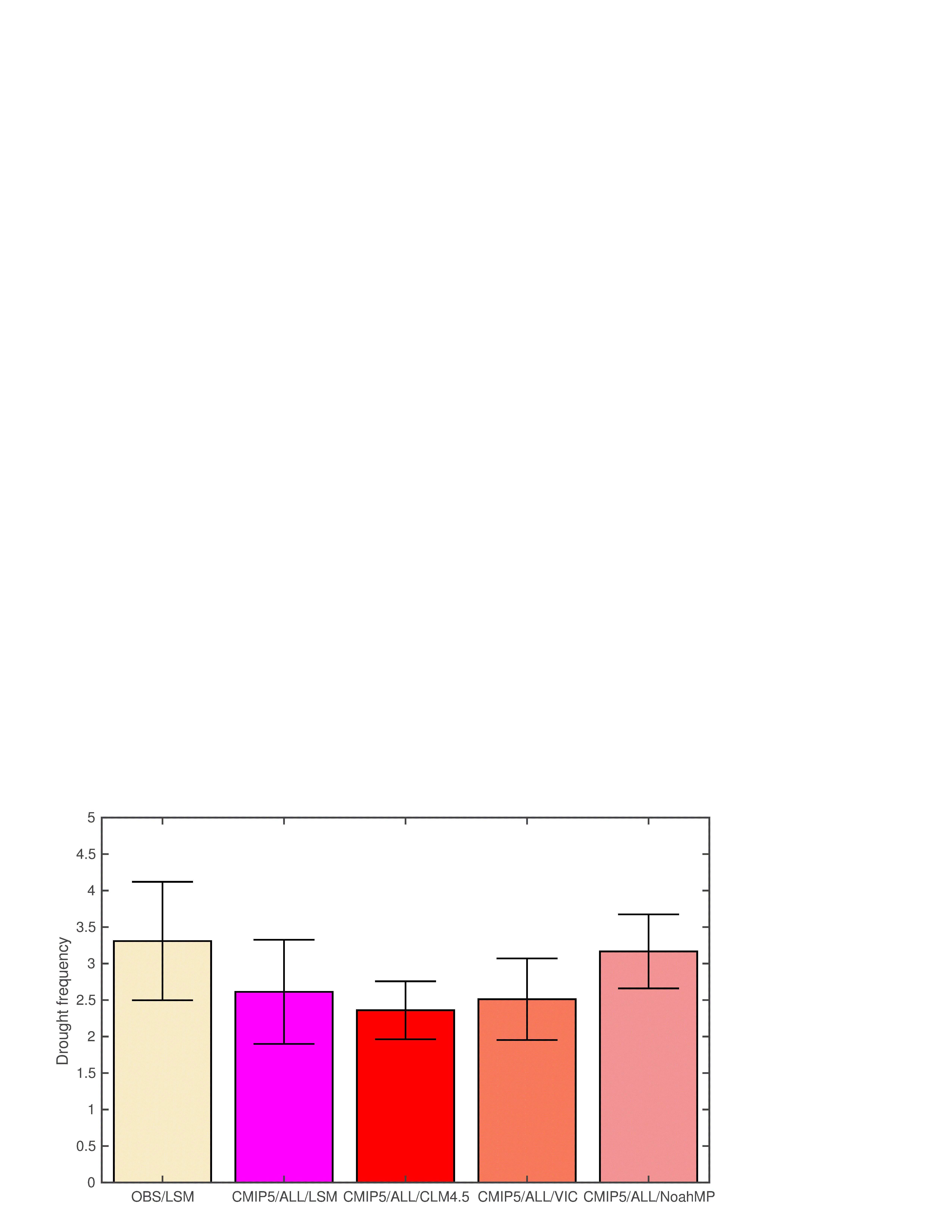


**Supplementary Figure 5.** The same as Supplementary Figure 4, but for the uncertainty ranges of flash drought frequency averaged over China. Here, OBS/LSMs represent LSM simulations driven by observed meteorological forcings during 1961-2005. The black bars show 5%-95% uncertainties.


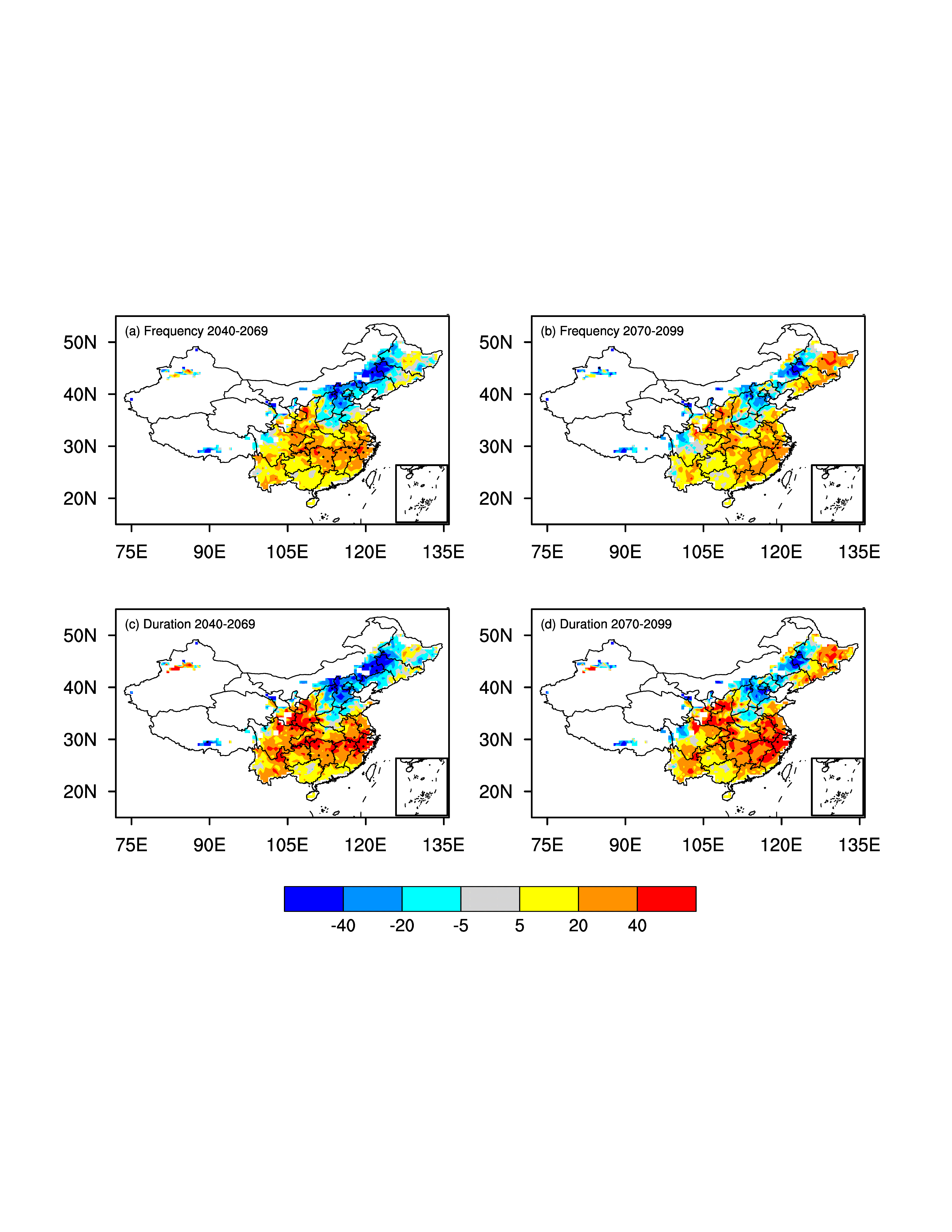


**Supplementary Figure 6.** The same as Figure 3, but for the relative changes (%) in frequency and duration of flash drought event. All the statistics were calculated during the growing seasons (April-September). Maps were created by using the NCAR Command Language (Version 6.3.0) [Software]. (2016). Boulder, Colorado: UCAR/NCAR/CISL/TDD. <http://dx.doi.org/10.5065/D6WD3XH5>. And the maps were updated with a database provided by <https://coding.net/u/huangynj/p/NCL-Chinamap/git/tree/master/database>


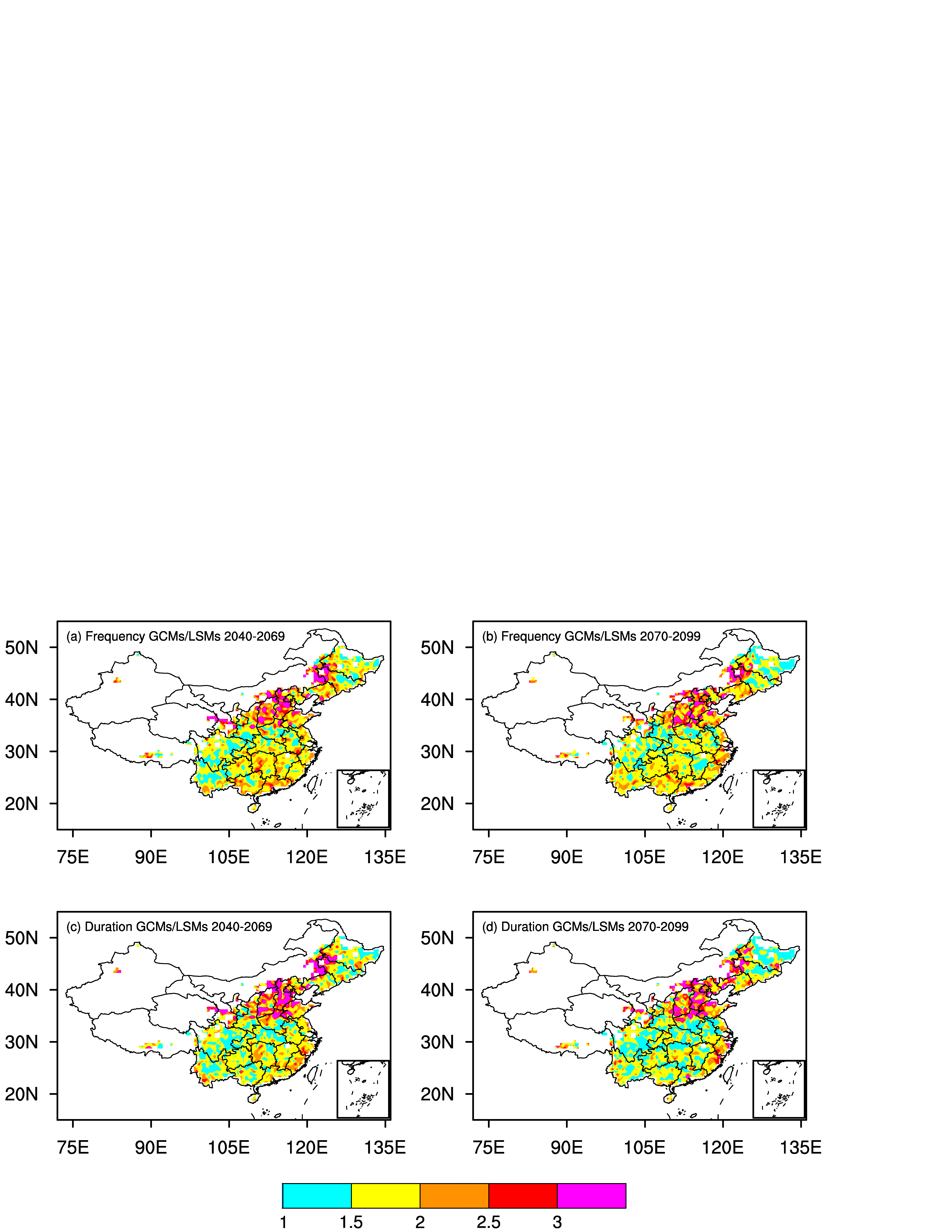


**Supplementary Figure 7.** The ratio between the Global Climate Model (GCM; 11 CMIP5 models used in this study) contribution and LSM contribution to the uncertainty ranges for the changes in flash drought frequency and duration shown in Supplementary Figure 6. The values larger than 1 suggest that the uncertainty from GCMs is larger than that from LSMs. Maps were created by using the NCAR Command Language (Version 6.3.0) [Software]. (2016). Boulder, Colorado: UCAR/NCAR/CISL/TDD. <http://dx.doi.org/10.5065/D6WD3XH5>. And the maps were updated with a database provided by <https://coding.net/u/huangynj/p/NCL-Chinamap/git/tree/master/database>


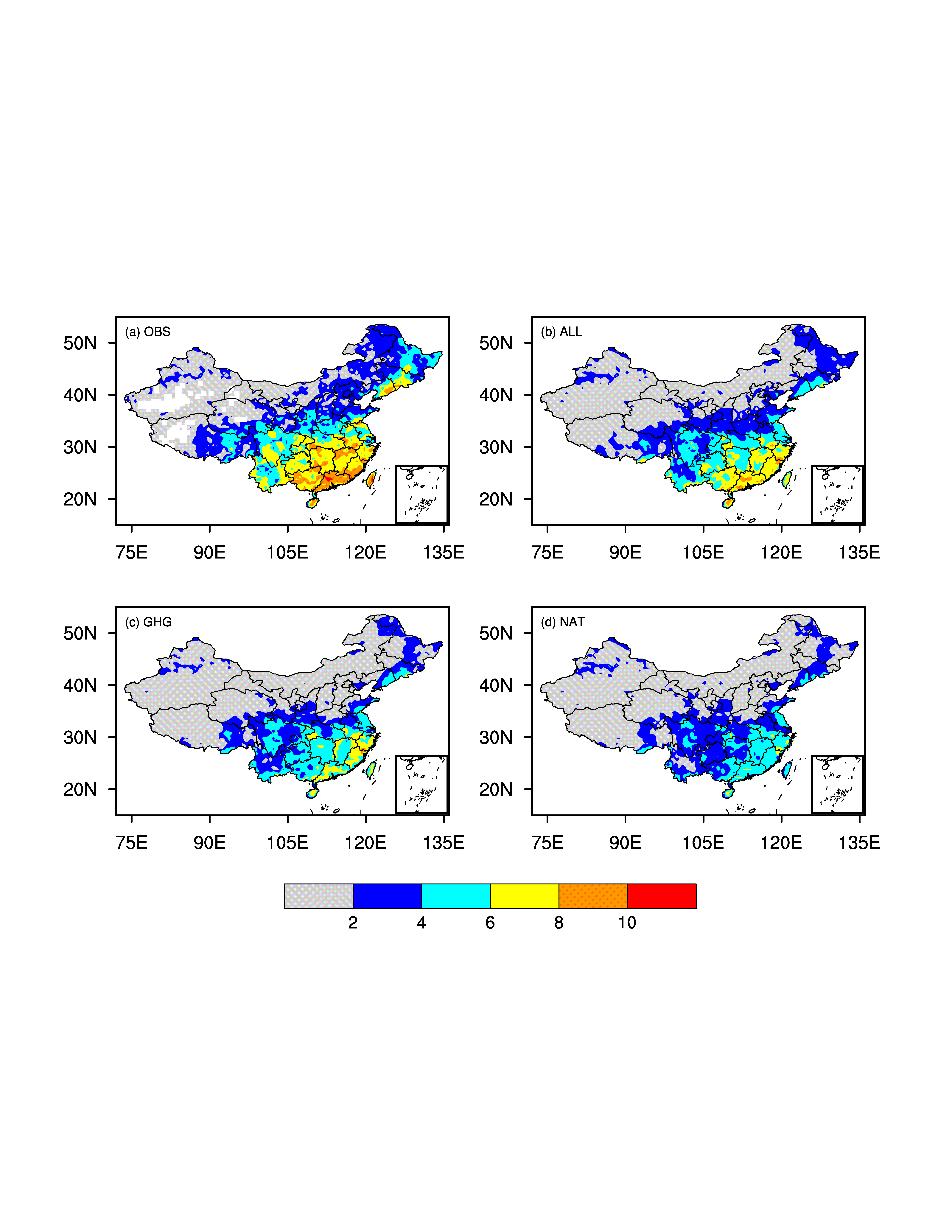


**Supplementary Figure 8.** Frequency of flash drought (events/decade) during the growing seasons (April to September) from (a) OBS/LSM ensemble simulations as well as (b)-(d) CMIP5/LSM ensemble simulations with ALL, GHG and NAT forcings, respectively. All statistics are based on the data during 1961-2005. Maps were created by using the NCAR Command Language (Version 6.3.0) [Software]. (2016). Boulder, Colorado: UCAR/NCAR/CISL/TDD. <http://dx.doi.org/10.5065/D6WD3XH5>. And the maps were updated with a database provided by <https://coding.net/u/huangynj/p/NCL-Chinamap/git/tree/master/database>


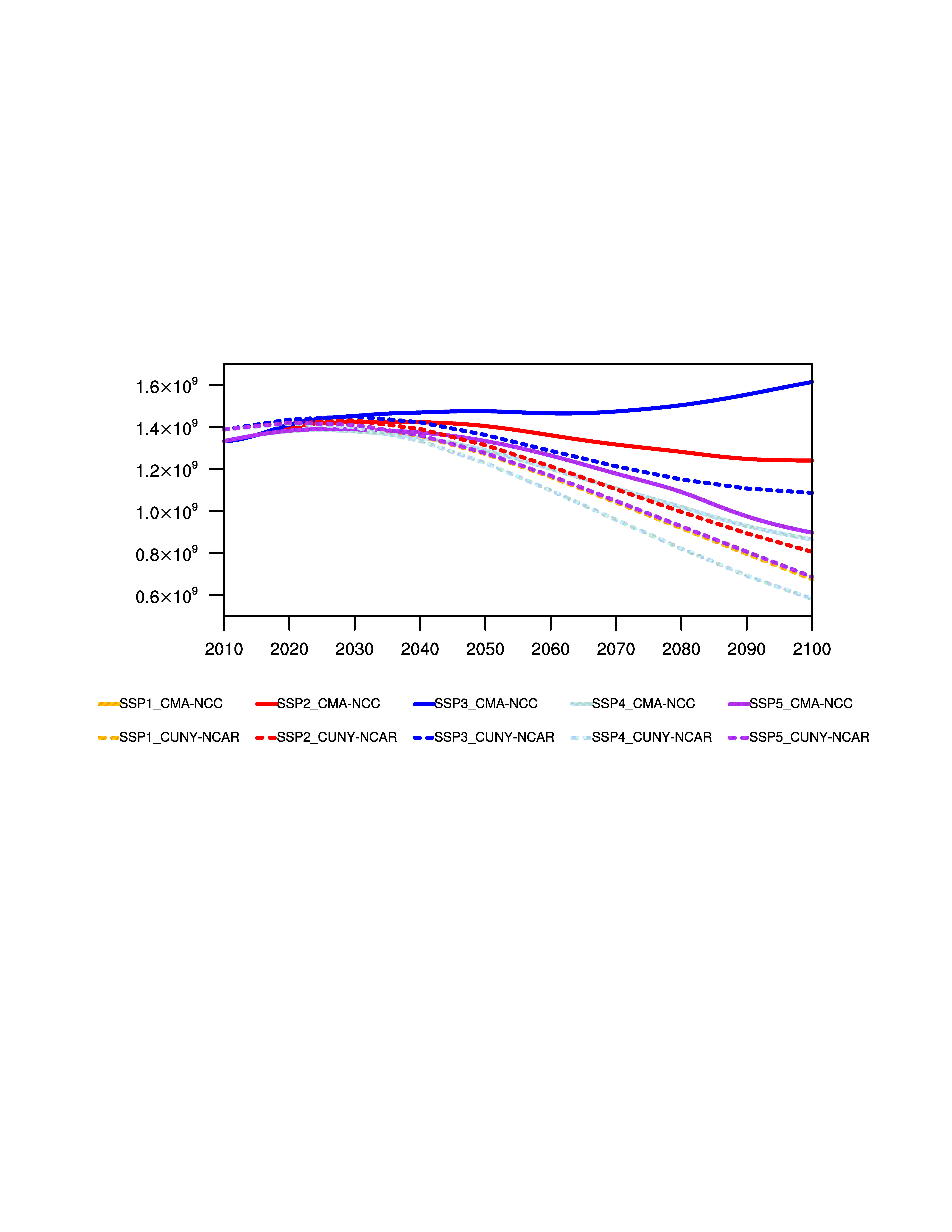


**Supplementary Figure 9.** Projected China population under SSPs 1-5 from CMA-NCC and CUNY-NCAR.


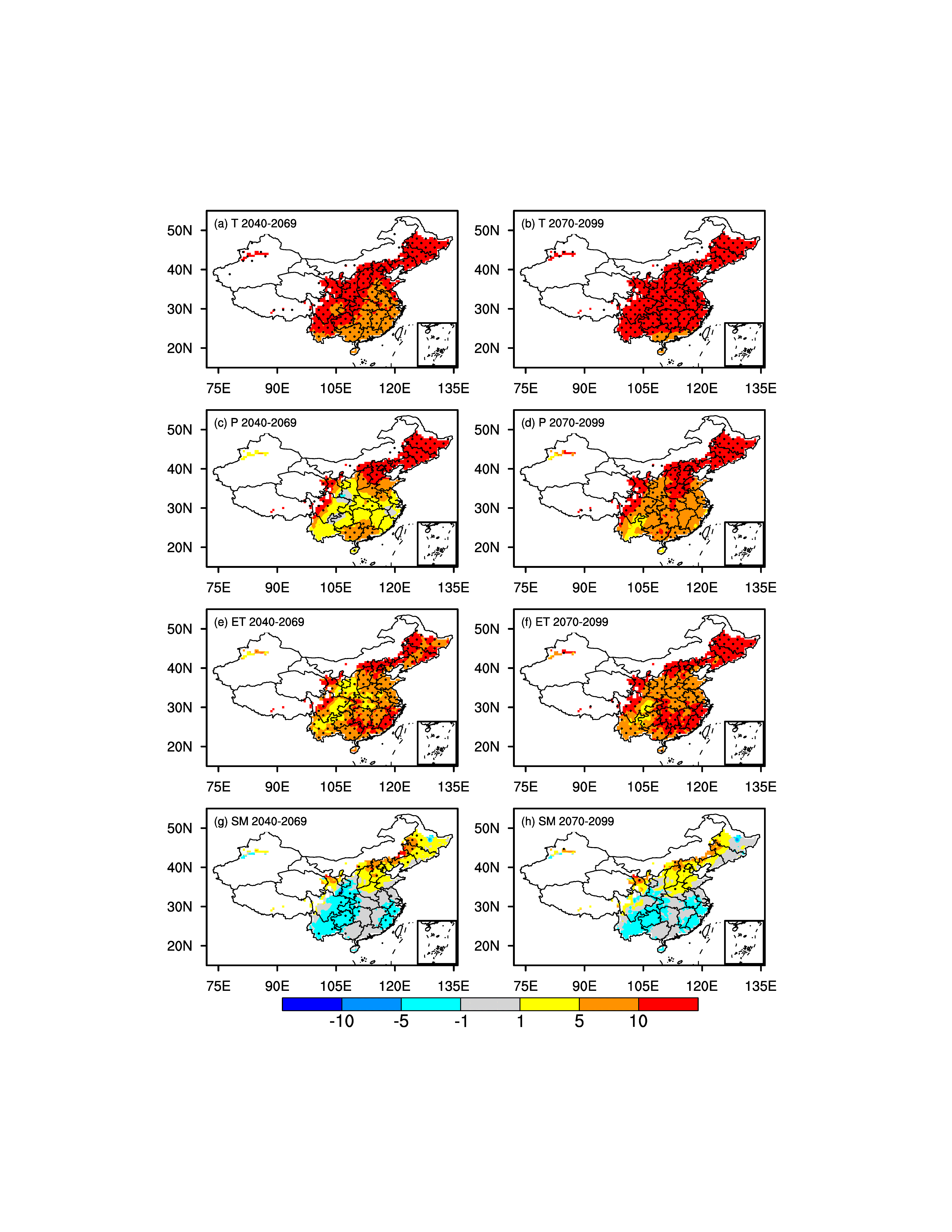


**Supplementary Figure 10.** The same as Figure 3, but for the relative changes (%) in April-September mean surface air temperature (T), precipitation (P), ET and soil moisture (SM). T and P are from 11 CMIP5 climate model simulations, and ET and SM are from 29 CMIP5/LSM combined simulations. Maps were created by using the NCAR Command Language (Version 6.3.0) [Software]. (2016). Boulder, Colorado: UCAR/NCAR/CISL/TDD. <http://dx.doi.org/10.5065/D6WD3XH5>. And the maps were updated with a database provided by <https://coding.net/u/huangynj/p/NCL-Chinamap/git/tree/master/database>


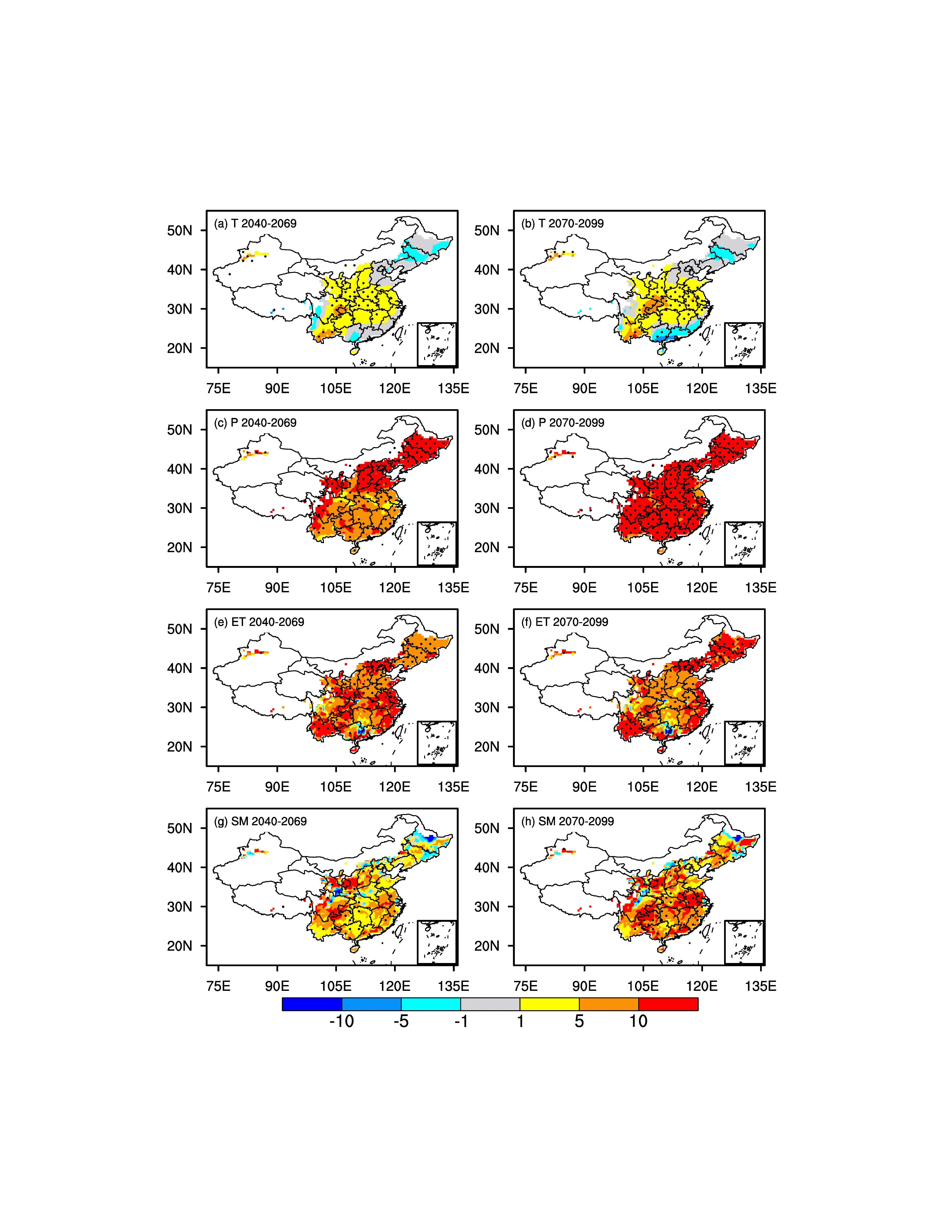


**Supplementary Figure 11.** The same as Figure 3, but for the relative changes (%) in variability (measured as standard deviation) of pentad (5-day) mean surface air temperature (T), precipitation (P), ET and soil moisture (SM). T and P are from 11 CMIP5 climate model simulations, and ET and SM are from 29 CMIP5/LSM combined simulations. All the statistics were calculated during the growing seasons (April-September). Maps were created by using the NCAR Command Language (Version 6.3.0) [Software]. (2016). Boulder, Colorado: UCAR/NCAR/CISL/TDD. <http://dx.doi.org/10.5065/D6WD3XH5>. And the maps were updated with a database provided by <https://coding.net/u/huangynj/p/NCL-Chinamap/git/tree/master/database>

**Supplementary Tables**

**Supplementary Table 1.** **CMIP5/LSM superensemble simulations.** CMIP5/ALL simulations were forced by both anthropogenic (greenhouse gases, aerosols, etc.) and natural (solar and volcanic activities) climate factors, CMIP5/GHG simulations were forced by anthropogenic greenhouse gases, CMIP5/NAT simulations were forced by natural factors only. CMIP5/CTL simulations were controlled to preindustrial situations, and were used to assess internal climate variability, where 96 sets of 45 years chunks were used in this study. All CMIP5 simulations were from the r1i1p1 realization, except for IPSL-CM5A-MR/GHG experiment where the r1i1p2 realization was used. Three land surface models (LSMs) are CLM4.5, VIC and NoahMP, which are represented by black, blue and red crosses respectively.

| CMIP5 Models | ALL  (1959-2005) | GHG  (1959-2005) | NAT  (1959-2005) | CTL | RCP4.5  (2006-2099) | RCP8.5  (2006-2099) |
| --- | --- | --- | --- | --- | --- | --- |
| CCSM4 | ×, ×, × | ×, ×, × | ×, ×, × |  | ×, ×, × | ×, ×, × |
| CNRM-CM5 | ×, ×, × | ×, ×, × | ×, ×, × | ×, ×, × | ×, ×, × | ×, ×, × |
| CSIRO-Mk-3-6-0 | ×, ×, × | ×, ×, × | ×, ×, × | ×, ×, × | ×, ×, × | ×, ×, × |
| GFDL-CM3 | ×, ×, × | ×, ×, × | ×, ×, × | ×, ×, × | ×, ×, × | ×, ×, × |
| GFDL-ESM2M | ×, ×, × | ×, ×, × | ×, ×, × | ×, ×, × | ×, ×, × | ×, ×, × |
| HadGEM2-ES | ×, ×, × | ×, ×, × | ×, ×, × | ×, ×, × | ×, ×, × | ×, ×, × |
| IPSL-CM5A-LR | ×, ×, × | ×, ×, × | ×, ×, × | ×, ×, × | ×, ×, × | ×, ×, × |
| IPSL-CM5A-MR | ×, × | ×, × | ×, × | ×, × | ×, × | ×, × |
| MIROC-ESM | ×, × | ×, × | ×, × | ×, × | ×, × | ×, × |
| MRI-CGCM3 | ×, × | ×, × | ×, × | ×, × | ×, × | ×, × |
| NorESM1-M | ×, × |  | ×, × |  | ×, × |  |
| Total | 29 | 27 | 29 | 24*4 | 29 | 27 |
